# Enorme tumeur de Buschk Loewenstein: à propos d'un cas historique

**DOI:** 10.11604/pamj.2014.18.38.3723

**Published:** 2014-05-10

**Authors:** Mourad Oussaid, Karim Ibn Majdoub Hassani

**Affiliations:** 1 Faculté de Médecine et de Pharmacie de Fès, Université Sidi Mohammed Ben Abdellah, Département de Chirurgie, CHU Hassan II Fès, Maroc

**Keywords:** Tumeur de Buschk Loewenstein, condylome acuminé, tumeur épithéliale, Buschke Loewenstein tumor, Condyloma Acuminata, epithelial tumor

## Image en medicine

La tumeur de buschk loewenstein (TBL) ou condylome acuminé géant est une MST à papilloma virus humain, elle est rare est toujours précédée du condylome acuminé. Cette tumeur épithéliale se caractérise essentiellement par son taux important de récidive, par son potentielle de transformation maligne et par son extension en profondeur. Nous présentons un cas d'un patient âgé de 52 ans, sans antécédents pathologiques notables, qui présente une TBL à localisation anale évoluant depuis plusieurs années. Cliniquement c'est une énorme formation péri anale faisant environ 17 cm de grand axe, ulcéro-bourgeonnante, macérée, nauséabonde, saignant au contacte. Une biopsie à été réalisée et le caractère condylomateux viral avec absence de signe de malignité a été confirmé sur l'histologie. L'IRM pelvienne a objectivé une extension au canal anale, à la graisse pelvi-réctale et en avant vers la racine de la verge (A, B, C). Nous avons réalisé une exérèse tumorale par voie périnéale emportant la quasi-totalité de la tumeur épargnant le sphincter anal et arrivant jusqu'au planché périnéal (D,E), les suites opératoires étaient simple. L’étude anatomopathologique de la pièce ne retrouve pas de territoire d'invasion ou de dégénérescence.

**Figure 1:**
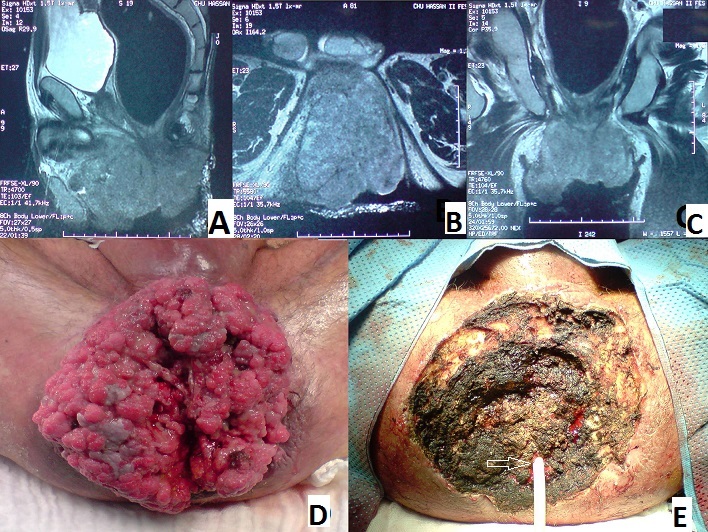
A) IRM en coupe sagittale montrant l'extension de la tumeur vers la racine de la verge; B) IRM en coupe axiale montrant la tumeur; C) IRM en coupe coronale montrant la tumeur; D) Aspect de la tumeur à l'examen du périnée du malade en position de taille périnéale; E) Aspect du périné après résection tumorale. (Flèche) Orifice anale catheterisé par une sonde de Foley

